# The Influence of Corticosteroids, Immunosuppressants and Biologics on Patients With Inflammatory Bowel Diseases, Psoriasis and Rheumatic Diseases in the Era of COVID-19: A Review of Current Evidence

**DOI:** 10.3389/fimmu.2021.677957

**Published:** 2021-07-08

**Authors:** Mengyuan Zhang, Xiaoyin Bai, Wei Cao, Junyi Ji, Luo Wang, Yang Yang, Hong Yang

**Affiliations:** ^1^ School of Medicine, Peking Union Medical College (PUMC), PUMC & Chinese Academy of Medical Sciences, Beijing, China; ^2^ Department of Gastroenterology, Peking Union Medical College Hospital, Peking Union Medical College & Chinese Academy of Medical Science, Beijing, China; ^3^ Department of Infectious Diseases, Peking Union Medical College Hospital, Peking Union Medical College, Chinese Academy of Medical Sciences, Beijing, China; ^4^ School of Medicine, Tsinghua University, Beijing, China; ^5^ Department of Respiratory and Critical Care Medicine, Peking Union Medical College Hospital, Peking Union Medical College & Chinese Academy of Medical Science, Beijing, China; ^6^ Department of Pharmacy, Peking Union Medical College Hospital, Peking Union Medical College & Chinese Academy of Medical Science, Beijing, China

**Keywords:** 2019-nCoV, COVID-19, SARS-CoV-2, autoimmune, corticosteroids, immunomodulators, biologics

## Abstract

Patients with inflammatory bowel disease, psoriasis or other rheumatic diseases treated with corticosteroids, immunomodulators and biologics might face additional risk during COVID-19 epidemic due to their immunocompromised status. However, there was still no unanimous opinion on the use of these therapy during COVID-19 epidemic. Current studies suggested that systemic corticosteroids might increase the risk of hospitalization, as well as risks of ventilation, ICU, and death among patients with immune-mediated inflammatory diseases. Anti-TNF agent was associated with lower rate of hospitalization, as well as lower risks of ventilation, ICU, and death. No significant changes in rates of hospitalization, ventilation, ICU and mortality were observed in patients treated with immunomodulators or biologics apart from anti-TNF agents. The underlying mechanism of these results might be related to pathway of antiviral immune response and cytokine storm induced by SARS-COV-2 infection. Decision on the use of corticosteroids, immunomodulators and biologics should be made after weighing the benefits and potential risks based on individual patients.

## Introduction

Since December 2019, the Coronavirus Disease 2019 (COVID-19) pandemic has spread around the globe and become a worldwide health threat. Patients infected by severe acute respiratory syndrome coronavirus 2 (SARS-CoV-2) might develop pneumonia, acute respiratory distress syndrome (ARDS), multiple organ failure and even death. Inflammatory bowel disease was considered to be associated with psoriasis and other rheumatic diseases like ankylosing spondylitis, as the prevalence of psoriasis and ankylosing spondylitis was significantly higher in patients with inflammatory bowel diseases and their first-degree relatives than control cohort, and vice versa (1–5). Some studies reported risk gene loci shared by inflammatory bowel disease psoriasis, and ankylosing spondylitis, including IL23R, IL12B, indicating shared pathophysiology of these diseases (6–9). Overall, the medications for these diseases were similar. Patients with immune-mediated inflammatory diseases mentioned above usually receive corticosteroids, immunosuppressants or biologics like anti-TNF agents, which can downregulate the patient’s immune response to some extent and in turn influence the clinical course and outcomes of COVID-19. A meta-analysis conducted by Shintaro et al. on Oct 2020 reported that the prevalence of COVID-19 among patients with immune-mediated inflammatory diseases was 0.011 (95% confidence interval [CI] 0.005-0.025), which was significantly higher than those in control group (odds ratio [OR] 2.19, 95%CI 1.05-4.58, p=0.04) (10). The incidence of hospitalization and mortality among patients with COVID-19 and immune-mediated inflammatory diseases was 0.35 (95%CI 0.23-0.50) and 0.066 (95%CI 0.036-0.12), respectively (10). However, the heterogeneity of studies included in the meta-analysis was considerable. Thus, more evidence is needed. It is worth cautioning that therapies based on immune suppression might prolong virus clearance and expose patients to adverse effects and superinfection. Additionally, immunocompromised patients might not display typical symptoms at the early phase of COVID-19, which could lead to a delay in intervention. On the other hand, therapies including corticosteroids, immunomodulators and biologics can induce and maintain remission of diseases including inflammatory bowel disease, psoriasis and other rheumatic diseases in these patients, making patients more resistant to virus invasion. Corticosteroids, immunomodulators, biologics could suppress inflammatory response, which might reduce the damage caused by COVID-19. In this review, we aimed to summarize the real-world data about the impact of corticosteroids, immunosuppressants and biologics on the clinical outcomes of COVID-19 among patients with inflammatory bowel disease, psoriasis and other rheumatic diseases. We searched on the database of Pubmed and Embase using terms (COVID-19 OR SARS-COV-2) AND (corticosteroids OR prednisone OR hydrocortisone OR dexamethasone OR immunomodulator OR immunosuppressant OR biologics) AND (autoimmune OR inflammatory OR rheumatic). The last search was performed on 20^th^ Dec 2020. Studies relevant to the purpose of our review was included. The references of included studies were also viewed.

## The Clinical and Pathological Characteristics of COVID-19

SARS-CoV-2 is a kind of coronavirus that spreads among people through respiratory droplets and close contact. According to the WHO Coronavirus Disease Dashboard, up to 22nd May 2021, the cumulative death rate of SARS-CoV-2 infection is 2.07% (3,437,545/165,772,430).

Patients infected with SARS-CoV-2 show symptoms of damage to multiple organs, including the lung, heart, gastrointestinal tract, and kidney. The most common symptoms of COVID-19 include fever, cough, myalgia, dyspnea, and diarrhea. Acute respiratory distress syndrome, heart failure, secondary infections, acute kidney injury, and shock are severe complications seen in COVID-19 (11). SARS-CoV-2 enters human cells by binding its S (spike) protein envelope to angiotensin-converting enzyme 2 (ACE2), which is present in type II alveolar cells, nasal mucosa, upper respiratory tract, endothelium, heart, kidney, and intestine cells (12, 13). SARS-CoV-2 could enter T cells through CD147 (14). SARS-CoV-2 induces cytokine storms in patients and leads to severe systemic reactions. SARS-CoV-2 induces cell death and the release of numerous chemokines that recruit immune cells. Proinflammatory mediators, including interleukin (IL)-1β, IL-6, IL-18, and TNF-α, are released and induce naïve T-cells to become Th1 or cytotoxic lymphocytes, which in turn secrete more cytokines. The immune complex of IL-6/IL-6R acts on gp130 and regulates MCP-1 and GM-CSF *via* the JAK-STAT pathway.

There might be concurrent macrophage activation syndrome driven by IL-1β, immunoparalysis (decreased HLA-DR on CD14 monocytes) and global lymphopenia driven by IL-6 in cases of COVID-19 (15). (14) TNF‐α also plays a part in lymphopenia. Diao et al. reported an association between higher serum levels of TNF‐α and lower lymphocyte counts (16). An *in vitro* experiment showed that TNF‐α could induce apoptosis of human T lymphocytes by binding to TNF‐RI (17). Lymphopenia leads to delayed viral clearance and, in turn, diversion of the adaptive immune response towards innate‐mediated inflammatory responses and cytokine storm, which ultimately leads to higher mortality from COVID-19 (18).

## Evidence on the Impact of Corticosteroids, Immunosuppressants and Biologics on Patients With Inflammatory Bowel Disease, Psoriasis, and Other Rheumatic Diseases

### Corticosteroids

**Figure 1:**
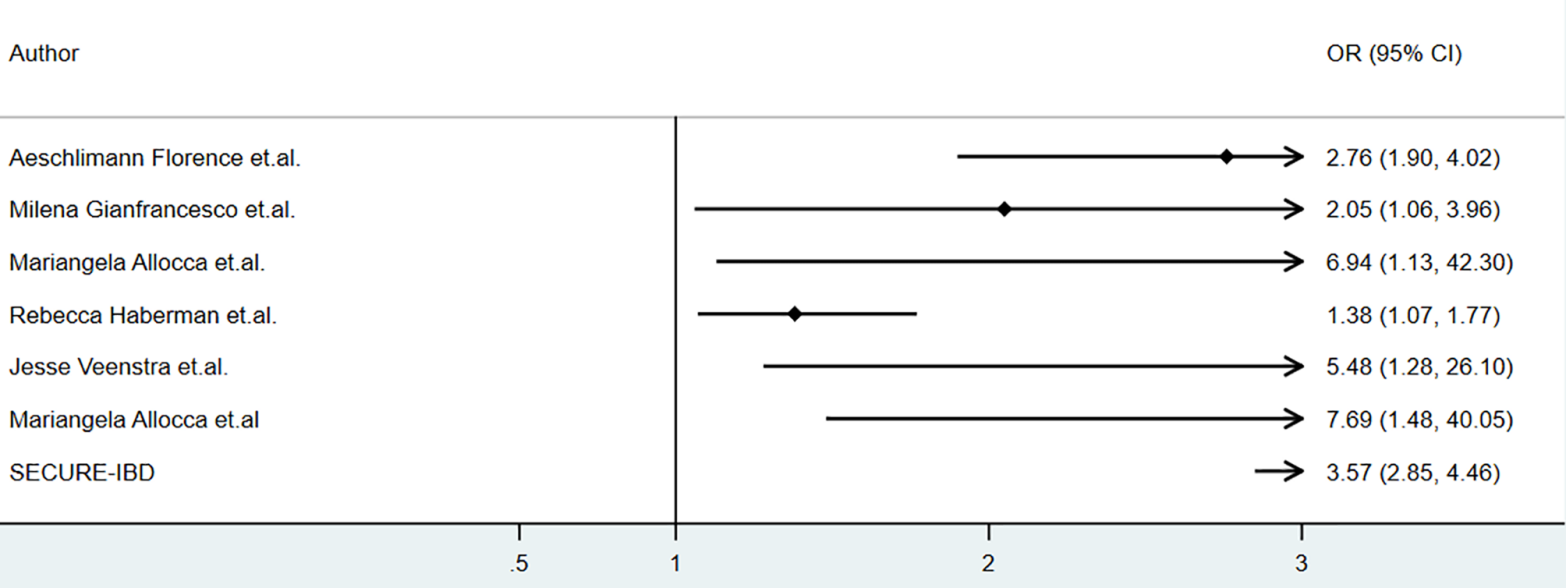
The association of corticosteroids and clinical outcomes of COVID-19 among patients with immune-mediated inflammatory disease (IMID).

Several studies reported the clinical outcomes of COVID-19 patients with inflammatory bowel disease, psoriasis and other rheumatic diseases receiving corticosteroids during COVID-19. The baseline characteristics of relevant studies were summarized in **Table 1**. The association of corticosteroids use and hospitalization was summarized as **Figure 1**.

**Table 1:** Baseline characteristics of cohort studies included.

First Author	Publication Date	Country	Sample size	Disease Type	Intervention	Antibiotic	Outcomes	Factors for Multivariate Analysis
Mariangela Allocca (19)	2020 Oct	Italy	41	29%UC, 22%CD, 20%PS, 10%PsA, 12%RA, 2%AS, 2%SSc, 2%SLE,1%others	7CS, 10IM, 28BIO	NA	hospitalizaiton, oxygen need, death,	age, gender, medication, comorbidities, rheumatic disease diagnosis
Milena Gianfrancesco (20)	2020 May	Multi-national	600	38%RA, 14%SLE, 12%PsA, 8%AS, 7%vasculitis, 5%SS, 3%SSc, 10%others	32CS	NA	hospitalizaiton, death,	age, gender, rheumatic disease diagnosis, comorbidities, medication
Anja Strangfeld (21)	2021 Jan	Multi-national	3279	36.7%RA, 12.5%AS, 12.6%PsA, 10.6%SLE, 7.7%vasculitis, 19.9%others	1056CS, 1267DMARD, 296IM, 1310BIO	NA	death	age, gender, rheumatic disease diagnosis, comorbidities, medication
FAIR/SFR/SNFMI/SOFREMIP/CRI/IMIDIATE consortium and contributors (22)	2021 Apr	France	694	30.7%RA, 23.8%AS, 10.1%PsA, 9.3%vasculitis,6.6%SLE, 3.6%SSc, 2.5%SS, 13.5%others	215CS, 328IM,354BIO	NA	moderate:hospitalization, severe:ICU or death	age, gender, diagnosis, medications, comorbidities
Jesse Veenstra (23)	2020 Dec	USA	77	33.8%RA/AS, 13.6%PS/PsA, 17.8%IBD, 15.5%SLE/DM/PM/MCTD/ILD/Scl	12CS, 41IM, 30BIO	NA	hospitalization,ventilator	NA
Rebecca Haberman (24)	2020 Apr	USA	86	16%PS, 24%PsA, 23%RA, 20%UC, 23%CD, 10%AS	62BIO,17MTX, 8CS	NA	hospitalizaiton, oxygen need, ICU, death,	age,sex,comorbidities,BMI,medication
Mariangela Allocca (25)	2020 Nov	Multi-national	97	44%UC,55%CD, 1% IBD-U	8CS,24IM,51BIO,	NA	hospitalization, ICU, death	age, gender, comorbidities, diagnosis, medication
Claudia Diniz Lopes Marques (26)	2021 Jan	Brasil	334	32.9%SLE, 28.4%RA, 13.5%AS, 6.9%SSc, 6.9%PsA, 3.3%vasculitis, 8.3%others	234CS, 154IM,116BIO	NA	hospitalization, ICU, ventilation, death	age, diagnosis, medication, comorbidities

UC, ulcerative colitis; CD, Crohn’s disease; IBD-U, inflammatory bowel disease-unclassified; PS, psoriasis; PsA psoriatic arthritis; RA, rheumatoid arthritis; AS, ankylosing spondylitis; SLE, Systemic lupus erythematosus; SS, Sjogren’s syndrome; SSc, systemic sclerosis; DM, dermatomyositis; PM, polymyositis; MCTD, mixed connective tissue disease; ILD, interstitial lung disease; CS, corticosteroids; IM, immunocmodulators; BIO, biologics; DMARD, disease-modifying antirheumatic drug; MTX, methotrexate.

As for the corticosteroids use in patients with inflammatory bowel disease. We retrieved data from SECURE-IBD on 18^th^ May 2021 and calculated the odds ratios, and the incidences of hospitalization (OR 3.57,95% CI 2.85-4.46, P<0.01), ICU (OR 4.80, 95% CI 3.23-7.01, P<0.01), ventilation (OR 3.94, 95% CI 2.45-6.15, P<0.01), and death (OR 5.45, 95% CI 3.30-8.74, P<0.01) were higher in patients who received oral/parenteral corticosteroids than those who did not (27). However, multivariate analysis was not conducted, as the data based on individual patients was not available. The potential influence by patients’ age, disease severity and comorbidities on results mentioned above could not be evaluated. Nonetheless, the result mentioned above was consistent with the report by Allocca, in which 97 patients with IBD were included, and treatment with corticosteroids was also associated with an increased risk of hospitalization (OR 7.69, 95% CI 1.48–40.05) at multivariable analysis (25). These results were consistent to a meta-analysis conducted by Anupam et.al., which showed that among patients with IBD, steroids increased the risk of hospitalization (RR 1.99, 95%CI 1.64–2.40; I^2 =^ 3%), need for ICU (RR 3.41, 95%CI 2.28–5.11; I^2 =^ 0) and mortality (RR 2.70, 95%CI 1.61–4.55; I^2 =^ 0) (28).

Corticosteroids was reported to be associated with higher risk of hospitalization among patients with COVID-19 and immune-mediated inflammatory diseases. According to a cohort of 694 patients with rheumatic and inflammatory diseases in France, corticosteroids was associated with higher risk of severe COVID-19 (adjusted odds ratio[aOR]=2.25, 95% CI: 1.33–3.79), hospitalization(aOR=2.76, 95% CI: 1.90–4.02,P<0.01) and mortality(aOR=2.64, 95% CI:1.36–5.12) (22).An analysis of data from the COVID-19 Global Rheumatology Alliance physician-reported registry using a multivariable-adjusted model showed that prednisone≥10 mg/day was associated with a higher risk of hospitalization (aOR 2.05, 95% CI 1.06 to 3.96) (20) and mortality(aOR1.69, 95%CI 1.18 to 2.41) (21). Mariangela Allocca reported a series of 41 patients with immune-mediated inflammatory disease (IMID), and corticosteroids was associated with a higher hospitalization rate (OR 6.94, 95%CI 1.13-42.3, P=0.03) and a higher rate of oxygen need(OR14.5, 95%CI 2.18−96.43) in univariate analysis, however, in multivariate analysis, comorbidities were the only independent risk factor for hospitalization and oxygen need (19). A cohort of 86 patients with IMID showed a higher incidence of hospitalization in those who received oral corticosteroids (adjusted OR 1.38, 95%CI 1.07-1.77) (24). A higher risk of hospitalization associated to corticosteroids was also seen in a cohort of 77 IMID patients (OR 5.48, 95% CI 1.28-26.1, P<0.05) but the result was not adjusted based on age, comorbidities, etc. (23) By results from ReumaCoV Brasil registry, methylprednisolone pulse therapy was associated with higher risk of hospitalization(prevalence ratio, PR 2.50; 95% CI 1.59 to 3.92; p<0.001) and ICU (PR 1.65; 95% CI 1.00 to 2.68; p<0.043) (25–28).

However, there are some limitations of these studies, including the study design, sample size, and follow-up. Data were collected from registries (which collect cases submitted by physicians) or case series, and the continuity of enrollment was not guaranteed. The baseline of enrolled patients was not balanced. Additionally, selection bias might exist, as reports mainly came from inpatient centers in epidemic areas, and patients with more severe disease were more likely to be reported. Moreover, confounding factors such as age, comorbidities, and concomitant drug use might to some extent explain the association between corticosteroids and clinical outcomes, and these factors were not evaluated in Veenstra’s reports.

For all of the limitations mentioned above, the impact of corticosteroids on the clinical outcomes of COVID-19 might be overestimated. Even so, researchers and organizations agreed that decisions on corticosteroid use should be made with caution in patients with autoimmune diseases. Guidelines by the British Society of Gastroenterology (BSG), American Gastroenterological Association (AGA), and the International Organization for the Study of Inflammatory Bowel Disease (IOIBD) recommended that systemic corticosteroids (especially prednisone>20 mg/d) should be tapered or switched to budesonide during the COVID-19 pandemic but should not be stopped at once (29–31). According to the American College of Rheumatology and German Society of Rheumatology, corticosteroids should be continued or initiated when needed, and the side effects should be monitored, especially when corticosteroids are used at high doses (32, 33). It is recommended that patients receiving corticosteroids be cautiously protected from infections by avoiding exposure to pathogens and optimizing nutrition and vaccination when needed.

### Immunomodulators

Immunomodulators seemed not be associated with altered risk of hospitalization. According to our analysis of data from SECURE-IBD, 6-MP/azathioprine (AZA) was associated with a higher risk of hospitalization (OR 1.47, 95%CI 1.16-1.85, P<0.01) and ICU(OR 1.87, 95%CI 1.16-2.90, P<0.01), and did not lead to significant change in risk of ventilation, and death(P>0.1) methotrexate (MTX) did not significantly alter the incidence of hospitalization, ICU, ventilation or death (27). A meta-analysis conducted by Anupam et al. confirmed no significant association between use of immunomodulators in IBD patients and risk of hospitalization (RR 0.89, 95%CI 0.37–2.10; I^2 =^ 83%), need for ICU (RR 0.71, 95%CI 0.17–3.02; I^2 =^ 45%) and mortality (RR 1.18, 95%CI 0.23–6.01; I^2 =^ 55%) (28).

Two study explored the impact of immunomodulators on the clinical outcomes of COVID-19 in patients with immune-mediated inflammatory diseases. Allocca et al. reported a cohort of 41 patients showed that immunosuppressants were not associated with altered risk of hospitalization and oxygen need (19). A cohort of 86 patients with IMID showed a higher risk of hospitalization in those who received methotrexate (adjustedOR 1.29, 95%CI 1.04-1.58) and no significant difference in risk of hospitalization in patients receiving azathioprine(adjusted OR 0.86 95%CI 0.43-1.69) (24).

Immunomodulators, including azathioprine (AZA), cyclophosphamide (CTX), and MTX, were recommended to be discontinued in patients with COVID-19 and not to be initiated during the pandemic. Evidence is still insufficient to demonstrate the influence of immunomodulators on COVID-19. Adverse effects of immunomodulators might affect the patient’s quality of life. One example was that AZA or CTX may cause lymphopenia, which was reported to be associated with mortality from COVID-19 (18).

Therefore, the use of immunomodulators should be evaluated based on individual cases. Physicians should weigh the side effects and the benefits of immunosuppressive drugs before making clinical decisions. As the pandemic interferes with normal medical care, the monitoring of adverse effects of immunomodulators should be optimized and individualized. This additional risk of unsurveillanced adverse effects should be cautioned by physicians.

### Biologics

**Figure 2:**
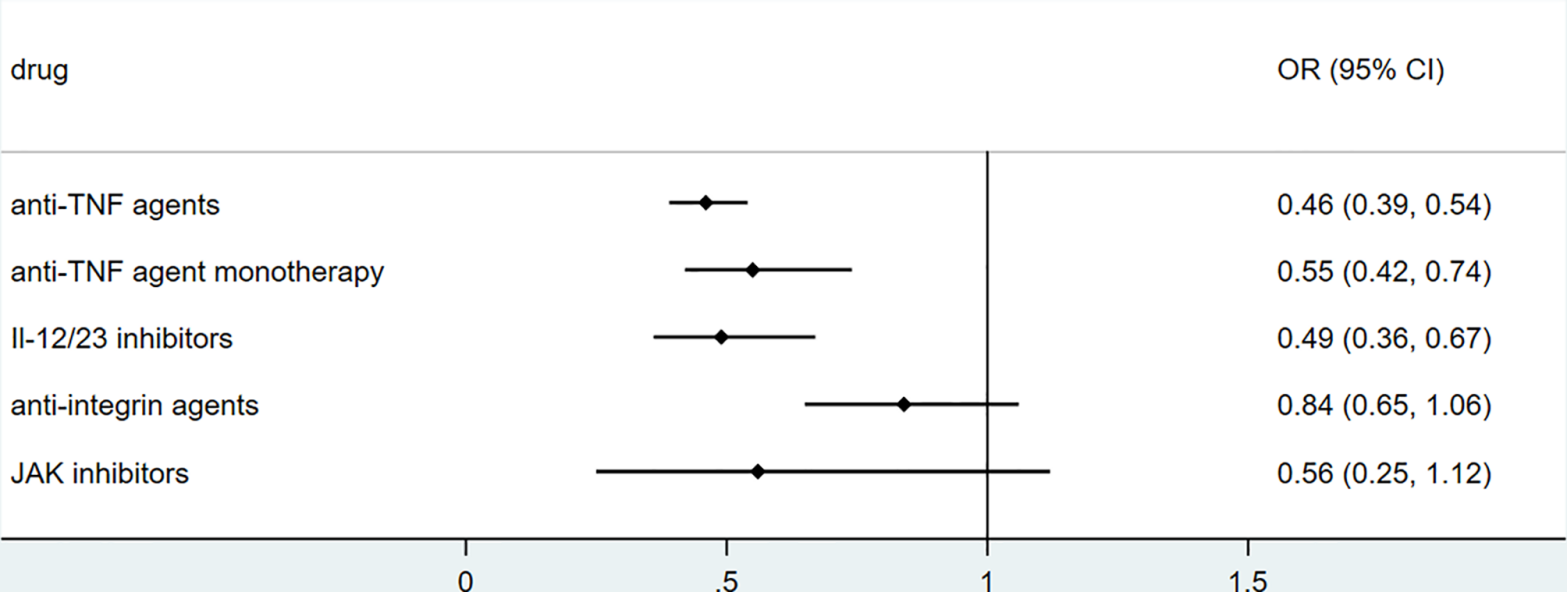
The association of biologics and hospitalization rate among IBD patients with COVID-19 according to data from SECURE-IBD (27). [The group ‘anti-TNF agent monotherapy’ was compared to the group receiving anti-TNF agent combined with 6MP/AZA/MTX. The other groups (‘Anti-TNF agents’, ‘IL-12/23 inhibitors’, ‘Anti-integrin agents’ and ‘JAK inhibitor’) were compared to those did not receive the corresponding biologics].

**Figure 3:**
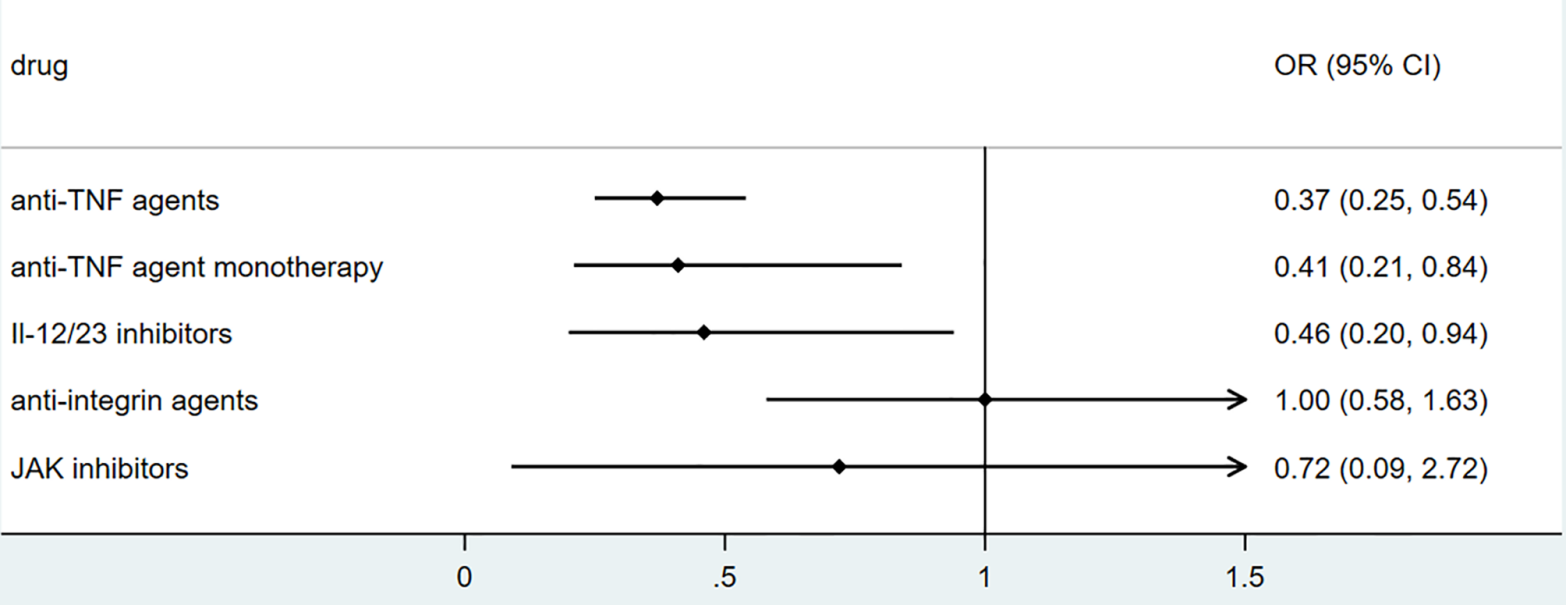
The association of biologics and ICU admission rate among IBD patients with COVID-19 according to data from SECURE-IBD (27). [The group ‘anti-TNF agent monotherapy’ was compared to the group receiving anti-TNF agent combined with 6MP/AZA/MTX. The other groups (‘Anti-TNF agents’, ‘IL-12/23 inhibitors’, ‘Anti-integrin agents’ and ‘JAK inhibitor’) were compared to those did not receive the corresponding biologics].

**Figure 4:**
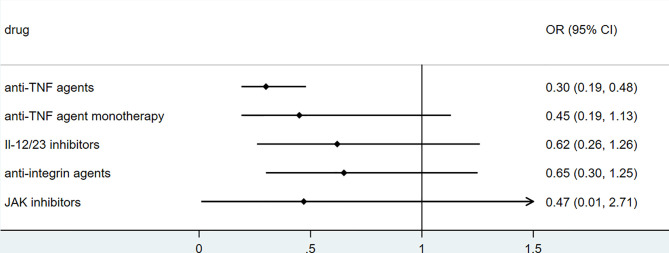
The association of biologics and ventilation rate among IBD patients with COVID-19 according to data from SECURE-IBD (27). [The group ‘anti-TNF agent monotherapy’ was compared to the group receiving anti-TNF agent combined with 6MP/AZA/MTX. The other groups (‘Anti-TNF agents’, ‘IL-12/23 inhibitors’, ‘Anti-integrin agents’ and ‘JAK inhibitor’) were compared to those did not receive the corresponding biologics].

**Figure 5:**
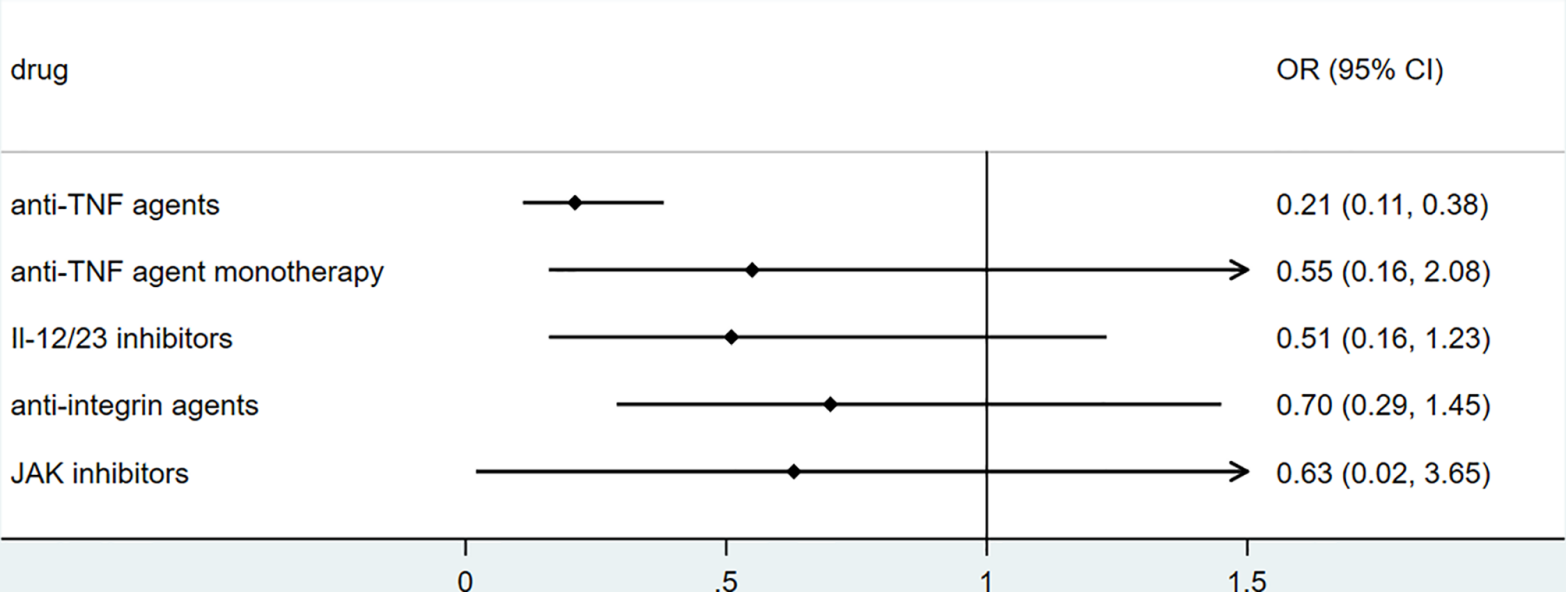
The association of biologics and mortality rate among IBD patients with COVID-19 according to data from SECURE-IBD (27). [The group ‘anti-TNF agent monotherapy’ was compared to the group receiving anti-TNF agent combined with 6MP/AZA/MTX. The other groups (‘Anti-TNF agents’, ‘IL-12/23 inhibitors’, ‘Anti-integrin agents’ and ‘JAK inhibitor’) were compared to those did not receive the corresponding biologics].

To explore the association between biologics used for treating inflammatory bowel disease, we performed secondary data analysis using published data from SECURE- IBD on 23^rd^ May 2021. The results were shown in **Figures 2**–**5**. We found that among IBD patients infected by COVID-19, anti-TNF agents were associated with lower incidences of hospitalization (OR 0.46, 95% CI 0.39-0.54, P<0.01), ICU (OR 0.37, 95% CI 0.25-0.54, P<0.01), ventilation (OR 0.30, 95% CI 0.19-0.48, P<0.01), and death (OR 0.21, 95%CI 0.11-0.38, P<0.01). The rate of hospitalization and ICU admission was lower in patients receiving anti-TNF-agent monotherapy than those treated with anti-TNF agents combined with 6MP/AZA/MTX (OR 0.55, 95%CI 0.42-0.74, P<0.01 and OR 0.37,95% CI 0.25-0.54, P<0.01, respectively). IL-12/23 inhibitors were associated with a lower risk of hospitalization (OR 0.49, 95%CI 0.36-0.67, P<0.01), and ICU (OR 0.46, 95%CI 0.20-0.94, P=0.03). Anti-integrin agents or JAK inhibitor did not significantly alter the risk of hospitalization, ICU, ventilation or death (27). However, from published data on the SECURE-IBD registry, we could not calculate the adjusted OR for each factor, as detailed information on individual patients was not available. We found that older age and comorbidities were significantly strong predictors for hospitalization/ICU/ventilation/death (27). The difference in the incidences of hospitalization/ICU/ventilation/death among patients treated with biologics might to some extent be due to the age distribution or comorbidities. The association between biologics and clinical outcome of COVID-19 should be verified by more studies.

**Figure 6:**
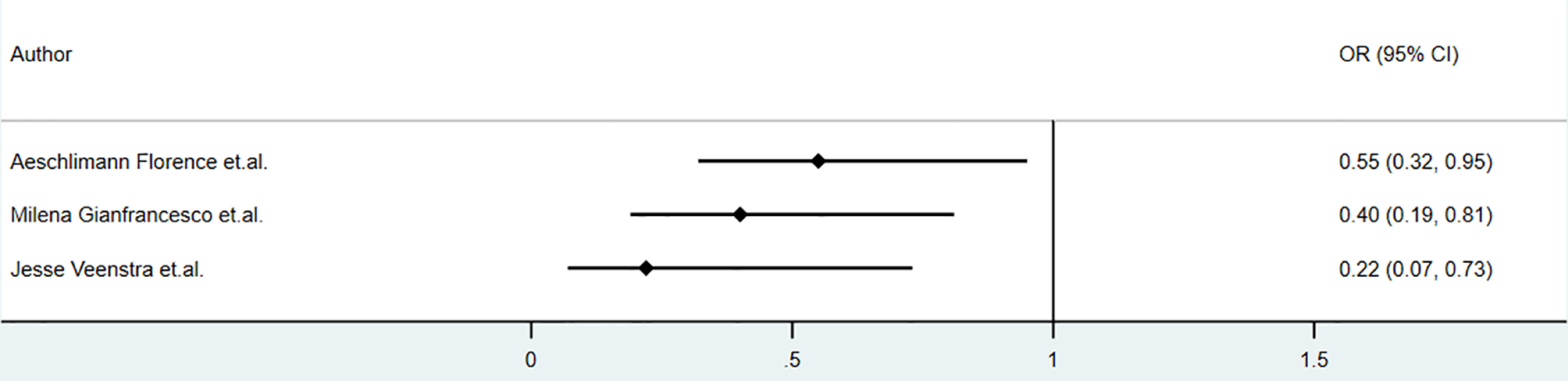
The association of anti-TNF agents and hospitalization of COVID-19 among patients with immune-mediated inflammatory diseases (IMID)

Biologic use in patients with psoriasis showed no significant correlation with the clinical outcome of COVID-19. Studies conducted by Damiani in May 2020 reported that in psoriasis patients, biologic treatment increased the risk of hospitalization (OR 3.59, 95%CI 1.49‐8.63, *P*<0.01), but it did not increase the risk of ICU admission(OR 3.41, 95%CI 0.21‐54.55, *P* =0.39) or death (OR 0.41, 95%CI 0.03‐6.59, *P* = 0.53) (34). The studies by Gisondi(5206 psoriasis, up to 1^st^ April 2020) and S.I. Cho et al.(7590 skin disease, up to 15^th^ May 2020) found no impact of biologics on clinical outcomes, including mortality, among patients with skin diseases (35, 36). The sample sizes of these studies were relatively large. However, the sample sizes of patients receiving biologics apart from anti-TNF and anti-IL-17 agents were relatively small. The studies mentioned above were retrospective or cross-sectional. Detailed information, including drug dosage, drug combination, and classification of psoriasis, was insufficient.Anti-TNF agents seemed to play a protective role in COVID-19 among patients with immune-mediated inflammatory diseases. The association between anti-TNF agents and hospitalization in patients with COVID-19 reported by some studies was shown in **Figure 6**. According to a cohort of 694 patients with rheumatic and inflammatory diseases in France(published in Dec 2020), use of anti-TNF agents did not increase the risk of severe COVID-19 (OR=0.44, 95% CI: 0.19–1.04) or death (OR=0.74, 95% CI: 0.22–2.01), and reduce the rate of hospitalization (OR=0.55, 95% CI: 0.32–0.95,P<0.01) (22). Analysis based on COVID-19 Global Rheumatology Alliance physician-reported registry showed that anti-TNF agents were associated with a lower risk of hospitalization (aOR 0.40, 95% CI 0.19-0.81) (20) and did not alter the mortality(aOR 0.85, 95%CI 0.52-1.36) (21), after controlling factors of age, sex, diagnosis, comorbidities and concomitant medication (20). Additionally, according to Veenstra et.al., anti-TNF agents decreased the risk of hospitalization (OR 0.22, 95% CI 0.07-0.73, P<0.05), but did not alter risk of ventilation(OR 0.61, 95%CI 0.05-4.49) and mortality(OR 0.75,95%CI 0.06-6.53) among patients with IMID, however these results were not adjusted and did not reflect the independent effect of anti-TNF agents on clinical outcomes (23). According to ReumaCoV Brasil registry, no use of tumor necrosis factor inhibitor (TNFi) was associated with higher risk of hospitalization(PR 2.51;95% CI 1.16 to 5.45; p=0.004) (26). These results were demonstrated in **Figure 3**. A meta-analysis conducted by Anupam et al. reported lower relative risk of hospitalization (RR 0.34, 95%CI 0.19–0.61; I^2 =^ 67%), need for ICU (RR 0.49, 95%CI 0.33–0.72; I^2 =^ 0) and mortality (RR 0.22, 95%CI 0.13–0.38; I^2 =^ 0) among IBD patients who were treated with biological agents (28).

The studies mentioned above indicated that anti-TNF agents decreased the risk of hospitalization. However, the limitations of these studies were as follows. The cohorts of these studies were mixtures of patients with different kinds of immune-mediated inflammatory disease, and the sample size of each disease was small. Additionally, information about the dosage and duration of medication was insufficient.

The studies mentioned above did not reach a unanimous conclusion about biologics use in patients with COVID-19 and autoimmune diseases. Biologics might increase the risk of severe COVID-19 and hospitalization in patients with skin diseases, which was not always the case. Interestingly, anti-TNF agents seemed to play a protective role in COVID-19, reducing the risk of hospitalization and ICU, ventilation or death. Anti-TNF agents could induce and maintain the remission of autoimmune disease and interfere with the pathway of cytokine storm. This might explain the results mentioned above.

Current data suggest that biologics (in addition to anti-TNF agents) do not influence mortality or hospitalization/ICU/ventilation rates. Biologics were proven to be of good efficacy and safety; thus, biologics therapy should not be stopped in patients with autoimmune diseases during the COVID-19 pandemic. More evidence is needed to confirm the efficacy and safety of anti-IL-17, anti-integrin, anti-IL-12/23 and JAKi in patients with COVID-19.

According to the BSG and AGA, TNF antagonists, anti-integrin, anti-IL-12/23 and Janus kinase inhibitors should be initiated as monotherapy or continued as prescribed in patients with IBD without SARS-CoV-2 (29, 30). For patients with IBD infected by SARS-CoV-2, delaying biologics (including anti-TNF, anti-IL-12/23, anti-integrin, and Janus kinase inhibitor) for 2 weeks was considered (29). The American College of Rheumatology and German Society of Rheumatology recommended that immunomodulators and biologics should be continued as prescribed in patients with inflammatory rheumatic diseases despite the SARS-CoV-2 pandemic (32, 33). For patients with psoriasis, the American Academy of Dermatology (AAD) and National Psoriasis Foundation (NPF) stated that there is currently insufficient evidence supporting the discontinuation of immunosuppressants in patients without COVID-19 infection. Patients with active COVID-19 infection should discontinue any immunosuppressive therapy (37, 38). In sum, decisions on biologics therapy should be made on an individual basis.

## Possible Explanations for the Impact of Drug Usage on COVID-19

### Corticosteroids

The impact of corticosteroids on the innate and adaptive immune systems was profound. The functions of lymphocytes (both T cells and B cells), mast cells, monocytes, eosinophils, basophils, neutrophils, macrophages, and antigen presenting cells were all affected by corticosteroids. The mechanism by which corticosteroids affect the clinical course of COVID-19 is debatable. Corticosteroids downregulate the release of cytokines, in turn reducing the damage caused by cytokine storms. In the period of SARS-CoV-1, methylprednisolone was known to reduce IL-8, MCP-1, IP-10, IL-6, IFN-γ (Th1 response), and IL-4 (Th2 response) (39). Corticosteroids, however, also led to delayed viral clearance. Nelson et al. reported that early corticosteroid treatment was associated with a higher plasma viral load of SARS-CoV-1 (40).

Corticosteroids seemed to increase the risk of hospitalization and oxygen need among patients with COVID-19 and autoimmune diseases. For the overall cohort of patients with COVID-19, some studies reported that corticosteroids reduced mortality among patients with COVID-19 (41, 42). One study reported prolonged virus clearance time and hospital stay, higher percentages of antibiotic therapy and severe disease associated with corticosteroids and no significant difference in mortality associated with corticosteroids (43). A meta-analysis of 7 randomized clinical trials conducted by REACT working group in Sep 2020 reported that corticosteroids could potentially reduce the 28-day all-cause mortality among patients with severe COVID-19 (44). The results of the meta-analysis were shown in **Table 2**.

**Table 2:** The association between use of corticosteroids and 28-day all-cause mortality among patients with COVID-19, according to meta-analysis by REACT working group.

Treatment (control: usual care or placebo)	Trials included	Patientsenrolled	Number of deaths	Fixed-effect Odds Ratio (95%CI, p-value)	Random-effects Odds Ratio (95%CI, p-value)
**Steroids (Overall)**	7	1703	647	0.66 (95%CI 0.53-0.82; P < 0.01)	0.70 (95% CI 0.48-1.01; P = 0.05)
**dexamethasone**	3	1282	527	0.64 (95%CI 0.50-0.82; *P* <0 .01)	0.65 (95% CI, 0.36-1.17)
**hydrocortisone**	3	374	94	0.69 (95% CI 0.43-1.12; *P* = 0.13)	0.87 (95%CI, 0.072-10.5)
**methylprednisolone**	1	47	26	0.91 (95% CI 0.29-2.87; *P* =0.87)	0.91 (95%CI, 0.29, 2.87; P = 0.87)
**low doses of corticosteroids**	4	1381	472	0.61 (95%CI 0.48-0.78; P <0 .01)	0.80 (95%CI, 0.063-10.32; P = 0.75)
**high-dose corticosteroids**	3	322	175	0.83 (95%CI 0.53-1.29; P = 0.46)	0.83 (95%CI, 0.53-1.29; P = 0.46)

It was considered that the ambiguous effect of corticosteroids might be partly due to the time window and dosage of corticosteroid use. Some studies reported reduced ICU/ventilation/death events among groups treated with early-phase corticosteroids (45, 46). However, the preliminary results of RECOVERY (NCT04381936) showing that in patients hospitalized with COVID-19, dexamethasone lowered 28-day mortality among patients under ventilation but not in those without oxygen need (47). However, data of RECOVERY was insufficient on domains like exclusion criteria, level of oxygen support and viral clearance. Additionally, most of patients did not receive remdesivir, an effective antiviral agent (48). Another trial, REMAP-CAP(NCT02735707), which focused on whether hydrocortisone improves outcome for patients with severe COVID-19, was halted early and did not reach a definitive conclusion (49). Another randomized clinical trial(NCT02517489) showed no significant change in the rate of death or persistent respiratory support when stopped early at 21 days (50). The CoDEX Randomized Clinical Trial(NCT04327401) reported that dexamethasone plus standard care increased the number of days alive and free of mechanical ventilation over 28 days among patients with moderate to severe ARDS and COVID-19 compared to standard care alone (6.6 days vs 4.0 days) (51). However, CoDEX was an open-label clinical trial and 35% of patients received corticosteroids during the study period. A Cochrane systematic review and meta-analysis reported that corticosteroid used for influenza is associated with higher risk of infection and mortality, however, confounding factors was thought to influence the results of studies (52). Nevertheless, more studies are needed to elucidate the role of corticosteroids in COVID-19.

### Anti-TNF-α Agents

One explanation of the association between anti-TNF agents and a lower risk of ICU and death was that TNF-α played a part in the pathological process of COVID-19, including the promotion of Th1 cells and the induction of lymphocyte apoptosis, which led to a worse prognosis of COVID-19. Huang reported that ICU patients with COVID-19 had higher plasma levels of TNF-α than non-ICU patients (53). Experiments on rats demonstrated that etanercept or TNF-α depletion could reduce inflammatory cell infiltration, inflammatory cytokine secretion and inflammation-induced lung damage, in turn reducing the severity and mortality of RSV or H1N1 pneumonia (54, 55). An ongoing clinical trial is evaluating the role of adalimumab in treating severe COVID-19 pneumonia (56). Another underlying reason for the better prognosis associated with the usage of anti-TNF agents might be the therapeutic effect of anti-TNF agents for primary diseases such as inflammatory bowel disease (57).

Previous studies demonstrated that TNF-α induced the production of glycosaminoglycans and collagen by pulmonary fibroblasts and myofibroblasts, which in turn led to pulmonary fibrosis (58). TNF-α along with IL-17A upregulated IL-8, which was released by the respiratory epithelium and led to focal neutrophilia that was associated with severe respiratory disease (59, 60).

These findings suggested that by inhibiting TNF-α, cytokine storms might be suppressed. Additionally, some experiments on animals showed that anti-TNF agents reduced inflammation-induced lung damage and mortality in rats with RSV or H1N1 pneumonia (54, 55). Anti-TNF agents are regarded as both recommended therapies for IBD and potential therapies for cytokine storms caused by COVID-19.

### JAK Inhibitors

Tofacitinib, an effective oral JAK2/1/3 inhibitor, and baricitinib, a selective JAK1/JAK2 inhibitor, were approved by the FDA for the treatment of rheumatoid arthritis (RA) and ulcerative colitis(UC). Baricitinib, a Janus kinase inhibitor, inhibits gp130 family cytokines (such as IL-6, IL-12, IL-23, and IFN-γ). Baricitinib could also inhibit AP2-associated protein kinase 1 (AAK1), which is a regulator of endocytosis, and in turn interrupt the entry of the virus into cells (61). This makes baricitinib a promising therapy for COVID-19-related cytokine storms. Tofacitinib can effectively block IL-2, IL-7, and IL-6 but cannot inhibit AAK1. However, no studies have reported the protective role of tofacitinib against COVID-19. Evidence from cohort studies is still insufficient to determine whether JAK inhibitors could reduce the severity of COVID-19.

### Anti-IL-17 Agents

The role of IL-17 in viral infection was intriguing and seemingly paradoxical, which might be due to the different roles of IL-17/IL-17R in different cells. On the one hand, IL-17 could enhance Th1 immune responses, promote cytotoxic T-cell activity, modulate antiviral B-cell activities, and induce protective inflammatory responses, thus hindering viral infections (62). On the other hand, IL-17 could also antagonize antiviral Th1 or cytotoxic lymphocytic responses, enhance the survival of virus-infected cells, and promote virus replication, which promotes viral infections (62). Moreover, a meta-analysis found a higher risk of respiratory tract infection in patients receiving anti-IL-17 agents compared to placebo (OR 1.56, 95% CI 1.04-2.33) (63). However, there was no evidence of a higher risk of SARS-CoV-2 infection associated with anti-IL-17 agents. IL-17 could also contribute to the maintenance of tissue integrity, inhibit detrimental inflammation and mediate protective responses, and in turn limit viral infection-induced organ injury. However, IL-17A, along with TNF-α, stimulated the recruitment of neutrophils by inducing the release of IL-8, IL-6, IL-11, GM-CSF and G-CSF by respiratory epithelium, smooth muscle cells and fibroblasts (64, 65). In this way, IL-17 also induces excessive neutrophil migration and activation, promotes fibrosis development, antagonizes the development of Treg cells and induces Th2 immune responses, which contribute to viral infection-induced tissue pathology. Overall, the impact of IL-17 on the outcome of COVID-19 is still unclear.

### Immunomodulators

The commonly used immunomodulator methotrexate (MTX) can induce the apoptosis of activated T cells; downregulate the production of the proinflammatory factors TNF‐α, IL-6, and IL-8; and upregulate the anti-inflammatory cytokine IL-10. By reducing INF‐γ and IL‐2, methotrexate inhibited the Th1 response (66). It seemed that methotrexate could inhibit the excessive immune response related to COVID-19, but there was still no clinical evidence.

Cyclophosphamide (CTX) is a nonspecific antimetabolic drug that inhibits both humoral and cell-mediated immunity (67). Azathioprine (AZA) depresses the function of T cells, B cells, NK cells and antigen presenting cells (66). Cyclosporin A (CsA) reduced the production of IL-2 and INF‐γ and reduced the activity of NK cells (68). Cyclosporin A was reported to have broad-spectrum antiviral effects and to inhibit MERS-COV and SARS-COV replication in vitro (69). Pablo Guisado-Vasco et al. reported in Oct 2020 that among 607 patients with COVID-19, cyclosporine was associated with a significant decrease in mortality (OR 0.24, 95%CI 0.12-0.46; *p*<0·01) (70). However, evidence is still lacking for the role of CTX, AZA or CsA in COVID-19.

Thalidomide inhibits cyclooxygenase enzyme-2 (COX-2) and downregulates prostaglandin E2 (PGE2), TNF-α, IL-1, and IL-6 (71). This indicates that thalidomide might be a potential therapy for COVID-19. Chen et al. reported a case of COVID-19 pneumonia recovered by thalidomide combined with low‐dose short‐term glucocorticoid (72). Two ongoing clinical trials (NCT04273581 and NCT04273529) explored the efficacy and safety of thalidomide in treating COVID disease.

There are still no cohort studies reporting the associations observed between immunomodulators (MTX, CsA, CTX, AZA, and thalidomide) and clinical outcomes of patients with COVID-19 and immune-mediated inflammatory diseases. Benefits and risks need to be carefully weighed before making decisions about immunomodulator use.

## Summary

In this review, we summarized the impact of corticosteroids, immunomodulators and biologics on the outcome of COVID-19 in patients with autoimmune diseases. Current data from retrospective cohorts suggested that systemic corticosteroids, especially at higher dosages, were associated with a higher risk of hospitalization, ICU admission, ventilation and higher mortality, which was based on multivariate analysis regarding age, gender, comorbidities and diagnosis of autoimmune diseases. In some cohorts, anti-TNF agents were associated with a lower risk of hospitalization, ICU admission, ventilation and death. No statistically significant difference in hospitalization/ICU/ventilation/mortality rate was observed between patients receiving immunomodulators (e.g., MTX, AZA) or biologics apart from anti-TNF agents (anti-integrin, anti-IL-17, anti-IL-12/23, JAKi) and those who did not.

As the clinical data of COVID-19 combined with immune-mediated inflammatory diseases are still insufficient and scattered, it is difficult to draw convincing conclusions. First, the sample size of studies included in this review was limited, and might not be sufficient to draw a strong conclusion. Second, the severity of inflammatory bowel disease, psoriasis or other rheumatic diseases, and concomitant antibiotics were not analyzed in these studies, which was considered as confounding factors. Strong evidence is needed to prove the impact of corticosteroids, immunomodulators and biologics on the clinical course of COVID-19 and guide the clinical application of drugs during the pandemic.

## Author Contributions

MZ, XB, WC, and HY conducted the serach and selection of related published articles. MZ, XB, and JJ participated in reading the articles and retrieving data. WC, LW YY, and HY gave comments and guide modification of contents. All authors contributed to the article and approved the submitted version.

## Funding

This work was supported by National Natural Science Fund (81970495, 81570505), Beijing Natural Science Fund (7202161), and the CAMS Innovation Fund for Medical Science (2016-I2M-3-001, 2019-I2M-2-007). The funding source had no involvement in study design, collection, analysis and interpretation of data, writing of the report or the decision to submit the article for publication.

## Conflict of Interest

The authors declare that the research was conducted in the absence of any commercial or financial relationships that could be construed as a potential conflict of interest.
